# Supplementary material for: Molecular Docking Study on Several Benzoic Acid Derivatives against SARS-CoV-2
Source: Molecules. 2020 Dec 10;25(24):5828. doi: 10.3390/molecules25245828 (PMC7770597; doi:10.3390/molecules25245828)
Supplement: Supplementary file 1 [file molecules-25-05828-s001.pdf]

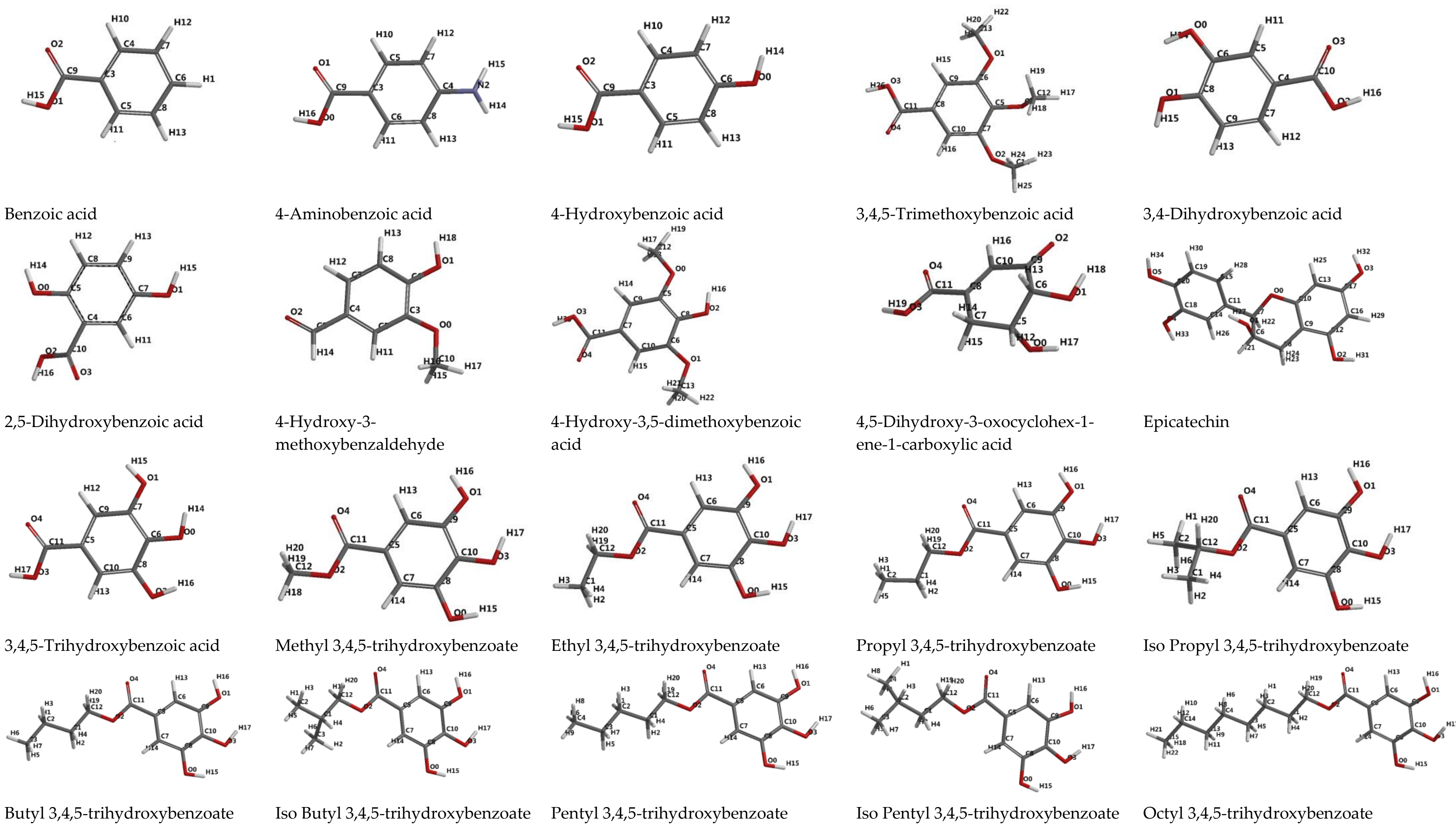

Figure S1. 3D optimized structures of investigated ligands and their numbering atomic labels.

Table S1. The list of intermolecular interactions between the compounds docked with 6WNP.

Ligand	Score	RMSD Å	Group interaction	Hydrogen Bond	Bond Length (Å)
USG A 101<br>(Boceprevir)	63.95	0.80	TYR54, CYS44, HIS41, THR25, THR26, LEU27, MET49, ARG188, ASP187, VAL186, GLN189, THR190 ALA191, GLN192, PRO168, LEU167, GLU166, MET165, HIS165, CYS145, GLY143, SER144, HIS163, LEU141, PHE140, ASN142	O sp^2 (O01) - N sp^2 CYS 145<br>O sp^2 (O01) - N sp^2 SER 144<br>O sp^2 (O01) - N sp^2 GLY 143<br>O sp^3 (O04) - N sp^2 HIS 41<br>N sp^2 (N11) - O sp^2 HIS 164<br>O sp^2 (O35) - N sp^2 GLU 166<br>N sp^2 (N27) - O sp^2 GLU 166<br>N sp^2 (N29) - O sp^2 GLU 166	2.900<br>3.053<br>2.783<br>2.604<br>3.103<br>3.118<br>2.908<br>3.229
Benzoic acid	29.59	0.09	TYR54, HIS41, MET49, CYS145, HIS164, MET165, ASP187, ARG188, GLN189, VAL186, PHE181	O sp^3 (O1) - O sp^2 HIS164<br>O sp^3 (O1) - N sp^2 HIS41	2.977<br>3.096
4-Aminobenzoic acid	31.37	0.23	ASN142, GLY143, LEU141, SER144, CYS145, PHE140, HIS164, MET165, GLU166, HIS163, HIS172	O sp^2 (O1) - N sp^2 GLY143<br>O sp^3 (O0) - O sp^2 ASN142<br>N sp^3 (N2) - O sp^2 PHE140<br>N sp^3 (N2) - O sp^2 GLU166<br>N sp^3 (N2) - N sp^2 HIS172	3.077<br>3.028<br>2.897<br>3.072<br>3.148
4-Hydroxybenzoic acid	31.34	0.007	ASN142, LEU141, GLY143, SER144, CYS145, PHE140, HIS164, HIS163, HIS162, MET165, GLU166	O sp^2 (O2) - N sp^2 GLU166<br>O sp^3 (O0) - O sp^3 SER144<br>O sp^3 (O0) - N sp^2 CYS145<br>O sp^3 (O0) - N sp^2 SER144<br>O sp^3 (O0) - N sp^2 GLY143<br>O sp^3 (O0) - O sp^2 LEU141	2.674<br>2.912<br>3.119<br>2.737<br>2.958<br>2.651
3,4,5-<br>Trimethoxybenzoic<br>acid	33.06	0.73	GLN192, ALA191, THR190, VAL186, GLN189, ARG188, ASP187, MET49, PRO52, TYR54, HIS41, HIS164, MET165, GLU166, LEU167	O sp^3 (O3) - O sp^2 THR190<br>O sp^3 (O3) - N sp^2 GLN192<br>O sp^3 (O3) - N sp^2 ARG188<br>O sp^3 (O1) - N sp^2 ARG188	2.975<br>3.159<br>3.006<br>3.115
3,4-Dihydroxybenzoic<br>acid	34.76	0.07	ASN142, GLY143, LEU141, SER144, CYS145, PHE140, HIS163, HIS164, MET165, GLU166	O sp^2 (O3) - N sp^2 GLU166<br>O sp^3 (O0) - N sp^2 HIS163<br>O sp^3 (O0) - O sp^3 SER144<br>O sp^3 (O0) - O sp^2 LEU141<br>O sp^3 (O1) - O sp^2 LEU141<br>O sp^3 (O1) - O sp^3 SER144<br>O sp^3 (O1) - N sp^2 SER144<br>O sp^3 (O1) - N sp^2 GLY143<br>O sp^3 (O1) - N sp^2 CYS145	2.922<br>3.089<br>2.785<br>3.187<br>2.718<br>2.956<br>2.615<br>2.855<br>3.001
2,5-Dihydroxybenzoic<br>acid	33.84	0.01	HIS163, CYS145, SER144, GLY143, ASN142, LEU141, PHE140, GLU166, MET165, HIS172	O sp^2 (O3) - N sp^2 GLY143<br>O sp^3 (O2) - N sp^2 GLY143	3.046<br>2.873

				O sp^3 (O2) - N sp^2 SER144	2.613
				O sp^3 (O2) - O sp^2 LEU141	2.730
				O sp^3 (O2) - O sp^3 SER144	2.945
				O sp^3 (O2) - N sp^2 CYS145	2.981
				O sp^3 (O0) - O sp^2 LEU141	3.152
				O sp^3 (O0) - O sp^2 SER144	2.700
				O sp^3 (O0) - N sp^2 HIS163	3.114
4-Hydroxy-3-methoxybenzaldehyde	34.01	0.007	HIS41, CYS44, TYR54, PRO52, MET49, GLN189, ARG188, VAL186, ASP187, GLU166, MET165, PHE181, HIS164	O sp^3 (O0) - N sp^2 HIS41	2.835
				O sp^3 (O1) - O sp^3 TYR54	2.875
				O sp^3 (O1) - O sp^2 ASP187	2.967
4-Hydroxy-3,5-dimethoxybenzoic acid	37.25	0.02	HIS172, HIS163, GLU166, HIS164, MET165, HIS41, THR26, ASN142, GLY143, LEU27, LEU141, SER144, PHE140, CYS145	O sp^2 (O4) - N sp^2 GLU166	3.108
				O sp^3 (O1) - O sp^3 SER144	2.993
				O sp^3 (O3) - O sp^3 SER144	2.702
				O sp^3 (O3) - O sp^2 LEU141	2.417
				O sp^3 (O3) - N sp^2 SER144	2.646
				O sp^3 (O3) - N sp^2 CYS145	3.171
				O sp^3 (O3) - N sp^2 GLY143	2.969
				O sp^3 (O0) - N sp^2 GLY143	3.057
4,5-Dihydroxy-3-oxocyclohex-1-ene-1-carboxylic acid	32.36	0.11	PRO168, GLU166, MET165, LEU167, ARG188, GLN189, THR190, ALA191, GLN191, GLN192, ALA193	O sp^3 (O3) - O sp^2 GLN192	3.208
				O sp^3 (O3) - N sp^2 GLN192	2.835
				O sp^3 (O0) - O sp^2 THR190	2.912
				O sp^3 (O0) - N sp^2 GLN192	3.276
				O sp^3 (O1) - O sp^2 GLU166	3.059
Epicatechin	49.57	0.03	HIS41, MET49, ARG188, VAL186, GLN189, THR190, GLN192, LEU167, GLU166, HIS172, MET165, HIS164, PHE140, LEU141, HIS163, ASN142, SER144, CYS145, GLY143	O sp^3 (O5) - O sp^2 GLU166	2.631
				O sp^3 (O1) - N sp^2 HIS 41	3.035
				O sp^3 (O1) - O sp^2 HIS164	3.057
				O sp^3 (O4) - O sp^2 ASN142	2.862
				O sp^3 (O4) - N sp^2 GLY143	3.123
				O sp^3 (O5) - O sp^2 LEU141	3.186
				O sp^3 (O5) - O sp^3 SER144	2.829
				O sp^3 (O5) - N sp^2 HIS163	3.105
3,4,5-Trihydroxybenzoic acid	38.31	0.04	HIS172, PHE140, LEU141, HIS163, GLU166, SER144, CYS145, ASN142, GLY143, MET165, HIS164	O sp^2 (O4) - N sp^2 GLU166	3.117
				O sp^3 (O1) - N sp^2 HIS163	2.842
				O sp^3 (O1) - O sp^3 SER144	2.955
				O sp^3 (O3) - O sp^3 SER144	2.792
				O sp^3 (O3) - N sp^2 CYS145	3.181
				O sp^3 (O3) - N sp^2 SER144	2.719
				O sp^3 (O3) - N sp^2 GLY143	2.985
				O sp^3 (O3) - O sp^2 LEU141	2.506
				O sp^3 (O0) - N sp^2 GLY143	3.098

				O sp^3 (O0) - O sp^2 ASN142	2.718
Methyl 3,4,5-trihydroxybenzoate	39.34	0.13	PHE140, GLU166, MET165, HIS163, LEU141, SER144, CYS145, ASN142, GLY143	O sp^3 (O0) - N sp^2 HIS163	3.089
				O sp^3 (O0) - O sp^3 SER144	2.802
				O sp^3 (O0) - O sp^2 LEU141	3.187
				O sp^3 (O3) - O sp^2 LEU141	2.783
				O sp^3 (O3) - O sp^3 SER144	2.881
				O sp^3 (O3) - O sp^2 SER144	2.629
				O sp^3 (O3) - N sp^2 CYS145	2.899
				O sp^3 (O3) - N sp^2 GLY143	2.989
				O sp^3 (O1) - N sp^2 GLY143	3.107
				O sp^3 (O1) - O sp^2 ASN142	2.776
Ethyl 3,4,5-trihydroxybenzoate	39.61	0.07	LEU167, GLU166, MET165, LEU141, PHE140, ASN142, HIS163, CYS145, GLY143, SER144	O sp^3 (O2) - N sp^2 GLU166	3.149
				O sp^3 (O1) - O sp^2 ASN142	2.731
				O sp^3 (O1) - O sp^2 GLY143	3.123
				O sp^3 (O3) - O sp^2 GLY143	2.876
				O sp^3 (O3) - N sp^2 CYS145	2.984
				O sp^3 (O3) - N sp^2 SER144	2.616
				O sp^3 (O3) - O sp^3 SER144	2.943
				O sp^3 (O3) - O sp^2 LEU141	2.727
				O sp^3 (O0) - O sp^2 LEU141	3.303
				O sp^3 (O0) - O sp^2 HIS163	2.988
				O sp^3 (O0) - O sp^3 SER144	2.834
Propyl 3,4,5-trihydroxybenzoate	42.13	1.20	GLN189, MET49, GLU166, HIS172, MET165, HIS41, HIS164, ASN142, LEU141, PHE140, HIS163, LYS145, GLY143, SER144	O sp^2 (O4) - N sp^2 GLU166	2.928
				O sp^3 (O1) - N sp^2 HIS163	2.785
				O sp^3 (O1) - O sp^3 SER144	2.908
				O sp^3 (O3) - O sp^3 SER144	2.736
				O sp^3 (O3) - O sp^2 LEU141	2.450
				O sp^3 (O3) - N sp^2 SER144	2.675
				O sp^3 (O3) - N sp^2 GLY143	2.976
				O sp^3 (O3) - N sp^2 CYS145	3.176
				O sp^3 (O0) - N sp^2 GLY143	3.142
				O sp^3 (O0) - O sp^2 ASN142	2.907
Iso Propyl 3,4,5-trihydroxybenzoate	41.76	0.24	GLY189, MET49, HIS41, HIS164, MET165, GLU166, HIS172, HIS163, CYS145, SER144, PHE140, LEU141, GLY143, ASN142	O sp^2 (O4) - N sp^2 GLU166	2.801
				O sp^3 (O1) - N sp^2 HIS163	2.790
				O sp^3 (O1) - O sp^3 SER144	2.901
				O sp^3 (O3) - O sp^3 SER144	2.720
				O sp^2 (O3) - O sp^2 LEU141	2.435
				O sp^2 (O3) - N sp^2 SER144	2.662
				O sp^2 (O3) - N sp^2 GLY143	2.972
				O sp^2 (O3) - N sp^2 CYS145	3.174

				O sp^3 (O0) - N sp^2 GLY143	3.174
				O sp^3 (O0) - O sp^2 ASN142	3.008
Butyl 3,4,5-trihydroxybenzoate	45.09	1.51	HIS172, HIS163, PHE140, CYS145, SER144, LEU141, GLY143, ASN142, HIS164, GLU166, MET165, LEU167, VAL186, GLN192, ASP187, ARG188, THR190, GLN189	O sp^3 (O2) - N sp^2 GLU166	3.075
				O sp^3 (O0) - N sp^2 HIS163	2.813
				O sp^3 (O0) - O sp^3 SER144	3.044
				O sp^3 (O3) - O sp^3 SER144	2.683
				O sp^3 (O3) - O sp^2 LEU141	2.399
				O sp^3 (O3) - N sp^2 SER144	2.631
				O sp^3 (O3) - N sp^2 GLY143	2.966
				O sp^3 (O3) - N sp^2 CYS145	3.169
				O sp^3 (O1) - N sp^2 GLY143	2.930
				O sp^3 (O1) - O sp^2 ASN142	2.863
Iso Butyl 3,4,5-trihydroxybenzoate	45.39	0.06	GLN192, ALA191, PHE181, VAL186, THR190, ASP187, ARG188, TYR54, MET49, HIS41, GLN189, HIS1645, MET195, GLU166	O sp^3 (O0) - O sp^2 ARG188	2.685
				O sp^3 (O0) - N sp^2 GLN192	2.762
				O sp^3 (O0) - N sp^2 THR190	3.055
				O sp^3 (O0) - O sp^2 THR190	2.563
				O sp^3 (O3) - O sp^2 THR190	3.083
Pentyl 3,4,5-trihydroxybenzoate	48.77	0.93	HIS41, TYR54, MET49, ASP187, ARG188, GLN189, VAL186, PHE181, HIS172, GLU166, MET165, HIS164, HIS163, PHE170, LEU141, CYS145, SER144, ASN142, GLY143	O sp^2 (O4) - N sp^2 GLU166	3.108
				O sp^3 (O1) - N sp^2 HIS163	2.756
				O sp^3 (O1) - O sp^3 SER144	3.043
				O sp^3 (O3) - O sp^3 SER144	2.738
				O sp^3 (O3) - O sp^2 LEU141	2.451
				O sp^3 (O3) - N sp^2 SER144	2.676
				O sp^3 (O3) - N sp^2 GLY143	2.974
				O sp^3 (O3) - N sp^2 CYS145	3.177
				O sp^3 (O0) - N sp^2 GLY143	3.102
				O sp^3 (O0) - O sp^2 ASN142	3.033
Iso Pentyl 3,4,5-trihydroxybenzoate	46.17	46.17	THR190, ALA191, GLN192, GLN189, VAL186, ARG188, PHE181, ASP187, MET49, TYR54, HIS41, HIS164, MET1265, GLU166, LEU167, PRO168	O sp^3 (O0) - O sp^{2}ARG188	2.610
				O sp^3 (O0) - N sp^2 THR190	2.889
				O sp^3 (O0) - N sp^2 GLN192	2.794
				O sp^3 (O0) - O sp^2 THR190	2.470
				O sp^3 (O3) - O sp^2 THR190	3.203
				O sp^3 (O1) - O sp^2 GLU166	3.050
Octyl 3,4,5-trihydroxybenzoate	60.22	1.12	ASN142, GLY143, LEU141, SER144, PHE140, HYS163, CYS145, HYS164, MET165, GLU166, HYS41, PHE181, VAL186, ASP187, TYR54, ARG188, GLN189, MET49	O sp^3 (O0) - O sp^2 ASN142	2.762
				O sp^3 (O0) - N sp^2 GLY143	2.713
				O sp^3 (O3) - N sp^2 GLY143	2.980
				O sp^3 (O3) - O sp^2 LEU141	2.475
				O sp^3 (O3) - N sp^2 SER144	2.696
				O sp^3 (O3) - O sp^3 SER144	2.761
				O sp^3 (O3) - N sp^{2}CYS145	3.179

		O sp^3 (O1) - O sp^3 SER144	3.390
		O sp^3 (O1) - N sp^2 HIS163	2.849

Table S2. Quantum chemical reactivity parameters calculated with Koopman's relationships.

**Compound**	E_{HOMO}	E_{LUMO}	ΔE_{gap}	$I = -E_{HOMO}$	$A = -E_{LUMO}$	$\chi = (I + A)/2$	$\eta = (I - A)/2$	$\sigma = l / \eta$	$\mu = (E_{HOMO}+E_{LUMO})/2$	$\omega = \mu^2 / 2\eta$
Benzoic acid	-7.09	-1.31	5.78	7.09	1.31	4.20	2.89	0.35	-4.20	25.49
4-Aminobenzoic acid	-5.81	-0.80	5.01	5.81	0.80	3.31	2.51	0.40	-3.31	13.68
4-Hydroxybenzoic acid	-6.41	-1.04	5.37	6.41	1.04	3.73	2.69	0.37	-3.73	18.63
3,4,5-Trimethoxybenzoic acid	-6.14	-1.18	4.96	6.14	1.18	3.66	2.48	0.40	-3.66	16.61
3,4-Dihydroxybenzoic acid	-6.03	-1.04	4.99	6.03	1.04	3.54	2.50	0.40	-3.54	15.59
**2,5-Dihydroxybenzoic acid (gentisic)**	-5.77	-1.20	**4.57**	5.77	1.20	3.49	2.29	0.44	-3.49	13.88
4-Hydroxy-3-methoxybenzaldehyde	-5.95	-1.39	4.56	5.95	1.39	3.67	2.28	0.44	-3.67	15.35
4-Hydroxy-3,5-dimethoxybenzoic acid	-5.67	-0.97	4.70	5.67	0.97	3.32	2.35	0.43	-3.32	12.95
4,5-Dihydroxy-3-oxocyclohex-1-ene-1-carboxylic acid	-7.32	-2.70	4.62	7.32	2.70	5.01	2.31	0.43	-5.01	28.99
Epicatechin	-5.53	-0.15	5.38	5.53	0.15	2.84	2.69	0.37	-2.84	10.85
3,4,5-Trihydroxybenzoic acid	-5.99	-1.06	4.93	5.99	1.06	3.53	2.47	0.41	-3.53	15.31
Methyl 3,4,5-trihydroxybenzoate	-5.91	-0.94	4.97	5.91	0.94	3.43	2.49	0.40	-3.43	14.58
Ethyl 3,4,5-trihydroxybenzoate	-5.89	-0.91	4.98	5.89	0.91	3.40	2.49	0.40	-3.40	14.39
Propyl 3,4,5-trihydroxybenzoate	-5.88	-0.90	4.98	5.88	0.90	3.39	2.49	0.40	-3.39	14.31
Iso Propyl 3,4,5-trihydroxybenzoate	-5.88	-0.90	4.98	5.88	0.90	3.39	2.49	0.40	-3.39	14.31
Butyl 3,4,5-trihydroxybenzoate	-5.88	-0.90	4.98	5.88	0.90	3.39	2.49	0.40	-3.39	14.31
Iso Butyl 3,4,5-trihydroxybenzoate	-5.89	-0.91	4.98	5.89	0.91	3.40	2.49	0.40	-3.40	14.39
Pentyl 3,4,5-trihydroxybenzoate	-5.87	-0.89	4.98	5.87	0.89	3.38	2.49	0.40	-3.38	14.22
Iso Pentyl 3,4,5-trihydroxybenzoate	-5.88	-0.89	4.99	5.88	0.89	3.39	2.50	0.40	-3.39	14.29
**Octyl 3,4,5-trihydroxybenzoate**	-5.87	-0.89	**4.98**	5.87	0.89	3.38	2.49	0.40	-3.38	14.22

Table S3. Molecular properties for the investigated ligands, calculated with Spartan Software.

Compound	Score	logP	Mass	Area	Volume	PSA	Ovality	Polarizabilty	Dipole moment	ΔE_{gap}
Benzoic acid	29.59	0.79	122.123	145.82	127.07	33.690	1.19	50.31	1.92	5.78
4-Aminobenzoic acid	31.37	-0.93	137.138	159.37	137.39	58.471	1.24	51.33	3.92	5.01
4-Hydroxybenzoic acid	31.34	-0.29	138.122	157.71	134.14	53.444	1.22	50.98	1.84	5.37
3,4,5-Trimethoxybenzoic acid	33.06	-2.14	212.201	235.75	209.14	53.223	1.38	51.17	3.33	4.96
3,4-Dihydroxybenzoic acid	34.76	-1.37	154.121	162.40	141.06	71.217	1.24	51.63	4.60	4.99
2,5-Dihydroxybenzoic acid	33.84	0.81	154.121	162.42	141.23	71.262	1.24	51.75	4.62	4.57
4-Hydroxy-3-methoxybenzaldehyde	34.01	-1.53	152.149	176.32	154.40	41.012	1.27	52.82	3.15	4.56
4-Hydroxy-3,5-dimethoxybenzoic acid	37.25	-2.24	198.174	212.58	188.07	64.706	1.34	55.52	2.78	4.7
4,5-Dihydroxy-3-oxocyclohex-1-ene-1-carboxylic acid	32.36	-0.92	172.136	173.96	152.64	83.671	1.26	52.66	3.11	4.62
Epicatechin	49.57	-3.72	290.271	286.73	269.71	101.294	1.42	61.98	1.13	5.38
3,4,5-Trihydroxybenzoic acid	38.31	-2.46	170.120	170.22	148.00	89.408	1.26	52.21	2.41	4.93
Methyl 3,4,5-trihydroxybenzoate	39.34	-2.19	184.147	192.40	168.38	75.752	1.30	53.86	1.77	4.97
Ethyl 3,4,5-trihydroxybenzoate	39.61	-1.86	198.174	213.18	183.92	75.425	1.35	55.36	1.55	4.98
Propyl 3,4,5-trihydroxybenzoate	42.13	-1.37	212.201	233.37	205.33	75.433	1.39	56.85	1.45	4.98
Iso Propyl 3,4,5-trihydroxybenzoate	41.76	-1.54	212.201	232.50	205.35	75.068	1.38	56.85	1.54	4.98
Butyl 3,4,5-trihydroxybenzoate	45.09	-0.95	226.228	253.67	223.78	75.433	1.42	58.35	1.40	4.98
Iso Butyl 3,4,5-trihydroxybenzoate	45.39	-0.97	226.228	252.27	223.48	75.149	1.42	58.32	1.54	4.98
Pentyl 3,4,5-trihydroxybenzoate	48.77	-0.54	240.255	273.97	242.23	75.426	1.46	59.84	1.36	4.98
Iso Pentyl 3,4,5-trihydroxybenzoate	46.17	-0.62	240.255	217.72	241.83	75.415	1.45	59.81	1.40	4.99
Octyl 3,4,5-trihydroxybenzoate	60.22	0.72	282.336	334.86	297.59	75.390	1.55	64.34	1.31	4.98

* Units: Mass: $g \cdot mol^{-1}$; Area: Å^2; Volume Å^3; PSA (polar surface area): Å^2; Polarizability: $10^{-30} \cdot m^3$; Dipole moment: Debye; ΔE_{gap} ($E_{HOMO} - E_{LUMO}$): eV

Table S4. Correlation matrix of PCA*

	Score	logP	Mass	Area	Volume	PSA	Ovality	Polarizabilty	Dipole moment	Gap
Score	1.00	-0.03	0.91	0.91	0.92	0.57	0.92	0.97	-0.65	0.02
logP	-0.03	1.00	-0.26	-0.12	-0.13	-0.43	-0.07	-0.06	0.11	0.09
Mass	0.91	-0.26	1.00	0.95	0.98	0.62	0.95	0.93	-0.62	0.00
Area	0.91	-0.12	0.95	1.00	0.97	0.46	0.96	0.91	-0.62	0.02
Volume	0.92	-0.13	0.98	0.97	1.00	0.48	0.98	0.94	-0.64	0.03
PSA	0.57	-0.43	0.62	0.46	0.48	1.00	0.45	0.56	-0.30	-0.19
Ovality	0.92	-0.07	0.95	0.96	0.98	0.45	1.00	0.92	-0.63	-0.07
Polarizabilty	0.97	-0.06	0.93	0.91	0.94	0.56	0.92	1.00	-0.69	0.02
Dipole moment	-0.65	0.11	-0.62	-0.62	-0.64	-0.30	-0.63	-0.69	1.00	-0.44
Energy gap	0.02	0.09	0.00	0.02	0.03	-0.19	-0.07	0.02	-0.44	1.00

* r means the Pearson correlation coefficient
